# The Role of Diffusion-Weighted Imaging in the Evaluation of Treatment of Newly Diagnosed Type 2 Diabetic Patients

**DOI:** 10.7759/cureus.50712

**Published:** 2023-12-18

**Authors:** Hasan Gündoğdu, Uğur Avcı, Mustafa Başaran, Enes Gürün

**Affiliations:** 1 Radiology, Recep Tayyip Erdogan University, Rize, TUR; 2 Endocrinology, Recep Tayyip Erdogan University, Rize, TUR; 3 Radiology, Samsun University, Samsun, TUR

**Keywords:** insulin, apparent diffusion coefficient, diffusion-weighted imaging, pancreas, diabetes mellitus

## Abstract

Introduction: To compare the pre and post-treatment pancreatic apparent diffusion coefficient (ADC) values of type II diabetes patients with control subjects, and also to evaluate its effectiveness in evaluating the response to treatment.

Methods: The study included 35 newly diagnosed type 2 diabetic patients and 35 non-diabetic participants, matched for sex and age. Insulin and metformin treatment was given to the patients. Abdominal diffusion-weighted MR imaging was performed before and after the treatment. ADCs of the control group and patients pre and post-treatment were compared. In addition, the clinical parameters of the patients related to diabetes were recorded.

Results: There was a significant difference between the median pancreatic ADC values of the patients pre and post-treatment. While there was a significant difference between the median pancreatic ADC values of the patient and the control groups before the treatment, no significant difference after the treatment was observed. There was a positive correlation between mean pancreatic ADC values and age, as well as a negative correlation with Hb1Ac level and eGFR.

Conclusion: Pancreatic ADC values of newly diagnosed type II diabetes patients can be used as a marker of pancreatic function in the evaluation of response to treatment and clinical decisions.

## Introduction

Type 2 diabetes mellitus (T2DM) has increased exponentially in recent years due to increasing obesity, aging, and sedentary lifestyle and has become one of the leading causes of death worldwide [1]. In T2DM, pancreatic β-cells that are chronically exposed to hyperglycemia gradually deteriorate, and their β-cell mass decreases [2,3].

It has been suggested that pancreatic islet amyloid polypeptide deposits resulting from glucose toxicity have a significant impact on the pathogenesis of glucose intolerance, which may be associated with progressive loss of cell mass and pancreatic fibrosis [4]. Studies on pathological specimens have shown that islet amyloid polypeptide accumulation is a representative feature of the pancreas in patients with impaired glucose tolerance [5].

One of the best treatments for T2DM is insulin, as it can lower blood sugar without stimulating β-cells. In this way, β-cells do not need to produce and secrete large amounts of insulin during insulin therapy, and β-cells can rest [6]. Thus, the β-cell function is gradually restored, and β cells begin to function correctly. In studies, it has been reported that early intensive insulin therapy in subjects with newly diagnosed T2DM has a positive result in terms of preservation of β-cell function [7]. In addition, insulin therapy protects β-cells by suppressing apoptotic β- cell death, facilitating re-differentiation from endocrine progenitor cells to mature β-cells [8].

Metformin and insulin have been shown to improve β-cell function in adults with new-onset type 2 diabetes. It has been reported that the combination of insulin and metformin is superior to other combinations in terms of glycemic control, weight gain, and the frequency of hypoglycemia and that metformin is an effective adjunct to insulin in patients with T2DM [9]. Noninvasive imaging of the degree of pancreatic fibrosis before and after treatment in newly diagnosed T2DM patients treated with insulin + metformin may play a role in evaluating the response to treatment.

Diffusion-weighted imaging (DWI) is a technique developed based on the movements of randomly selected water molecules in the tissue and can be used to examine the structural properties of tissues. The apparent diffusion coefficient (ADC) value calculated from DWI is a quantitative parameter [10]. The amount of diffusion of the pancreas, chronic inflammation in the pancreas, and damage by fibrosis can be evaluated by DWI. It has been reported in the literature that ADC values are correlated with pancreatic fibrosis grades in pancreatectomy patients and decrease as the fibrosis grade increases [11]. In addition, a relationship between HbA1c values and ADC values has been shown. It has been stated that these results may reflect the histopathological changes in the pancreatic parenchyma and the clinical presentation of high HbA1c levels [12,13].

The study aims to compare pancreatic ADC values with control subjects before and after insulin + metformin treatment in patients with newly diagnosed T2DM and evaluate its effectiveness in evaluating the response to treatment.

This article was previously presented as an Oral Presentation at the V. International Health Science and Life Congress on March 12, 2022.

## Materials and methods

The study was performed with the approval of the local research ethics committee. All procedures were carried out in accordance with ethical rules and the principles of the Declaration of Helsinki. An informed consent form was obtained from the patients included in the study.

Patients diagnosed with T2DM according to the diagnostic criteria of the American Diabetes Association (ADA) and treated with insulin + metformin between September 2021 and February 2022 in the Endocrinology Clinic of our hospital were included in the study. In addition, control subjects without DM, hypertension, heart, and kidney disease matched with type 2 diabetes patients for sex and age were included in the study.

Exclusion criteria for both groups; being younger than 18 years old, HbA1c below 10, pancreatic mass, previous pancreatitis or pancreatic surgery, renal failure, malignant liver or adrenal mass affecting the pancreas anatomically and functionally, lack of diffusion-weighted images, missing laboratory data.

Demographic characteristics, medical history, and laboratory data of 35 diabetic patients and 35 healthy subjects included in the study were obtained from the hospital information system.

Magnetic resonance imaging (MRI) was performed with the patient supine, using a 3 Tesla MR scanner (Discovery w750, GE Healthcare, United States). Antecubital intravenous access was established before the examination, and a bolus injection of contrast material was administered intravenously, following the pre-contrast images. Conventional, dynamic, and DWI were obtained using standard abdominal coils. The parameters of the MRI sequences are presented in Table 1. After the images were obtained, they were recorded in our hospital’s PACS (Picture Archiving and Communication System).

**Table 1:** Parameters for MR imaging CE MRI: contrast-enhanced magnetic resonance imaging; DWI: diffusion-weighted imaging

Parameter	Axial T2W imaging	Coronal T2W imaging	In-phase/out-of-phase imaging	CE MRI	DWI (50, 800 s/mm^2^)
Echo time (m)	84	84	2.4/5.6	1.5	56
Repetition time (ms)	3768	3000	230	3.5	2700
Flip angle (degrees)	90	90	90	15	90
Intersection gap (mm)	1	1	1	-2.5	1
Slice thickness (mm)	5	5	5-6	5	5-6
Field of view (mm)	360–400	360–400	360–400	340–400	360–400
Matrix	320×224	288×192	256×192	288×224	128×128
Parallel imaging acceleration factor	2	-	2	2	2

The standard treatment of the patients was given as insulin + metformin. The treatment protocol was planned as metformin (2*1000 mg, total 2000 mg/day) + insulin (0.5 units/kg/day). Half of the total dose was administered subcutaneously as basal insulin (insulin glargine) and the other half as rapid-acting insulin (insulin aspart) divided into three meals. Pre-treatment MRI scans of the patients were performed on average 9 (min-max: 5-16) days after the start of treatment. DWI was repeated at least three months after the treatment (min-max: 3-5 months). Also, fasting blood glucose and HbA1c levels were checked after treatment.

An abdominal radiologist (H.G.), blinded to clinical information, reviewed all images on a workstation. Three separate region of interests (ROIs) (min-max: 30-40 mm^2^) were drawn on the pancreas's head, body, and tail on the ADC maps obtained from DWI images of the patients and control group, and the median ADC values were calculated for each part of the pancreas. ROI measurements were made from locations that matched the enlarged pancreatic duct or pancreatic parenchyma devoid of artifacts. The mean of the ADC values from the ROIs was considered the representative ADC of the pancreas and expressed as the median ± standard deviation of X × 10^−^^3^ mm^2^/s. ADC values of the patients before and after the treatment were compared with the control group. Additionally, the relationship between patients' ADC values and age, gender, HbA1c, and eGFR values was evaluated.

Statistical analyses were performed with the IBM SPSS Statistics, Version 23.0 (SPSS Inc., Chicago, USA) program. Descriptive statistics of the groups were reported as frequency and percentage (n, %). Continuous numerical variables were analyzed by normality analyses. Accordingly, those with normal distribution were reported as mean ± standard deviation, and those without normal distribution were reported as median (min-max). Difference analyses between groups were performed with the student's t-test or Mann-Whitney U test. Continuous variables before and after treatment were analyzed with the Wilcoxon Signed Ranked Test. Chi-square analysis was performed for the distribution of categorical data among the groups. Relationships between parameters were evaluated with Spearman correlation analysis and reported with rho coefficient. ROC curve analyses were performed to evaluate the parameters' diagnostic performance and determine the appropriate threshold values. Accordingly, area under the curve (AUC), sensitivity, and specificity values were reported. The limit of significance was accepted as p < 0.05.

## Results

The mean age of the patients included in the study was 54.9±9.1 (37-75) years. Twenty-seven (77%) of the patients were male, and eight (23%) were female. Age, gender, pre-treatment HbA1c, and other laboratory parameters of the patients are shown in Table 2.

**Table 2:** Demographic characteristics and laboratory parameters of diabetic patients eGFR: estimated glomerular filtration rate

	Diabetic Patients	
	Mean	Standard Deviation
Age (years)	54.86	9.14
HbA1c (%)	12.69	1.87
eGFR (mL/min/1.73 m^2^)	85.49	12.69
Cholesterol (mg/dL)	242.29	50.32
Glucose (mg/dL)	313.43	77.28
Hemoglobin (g/dL)	14.79	2.49

**Figure 1:**
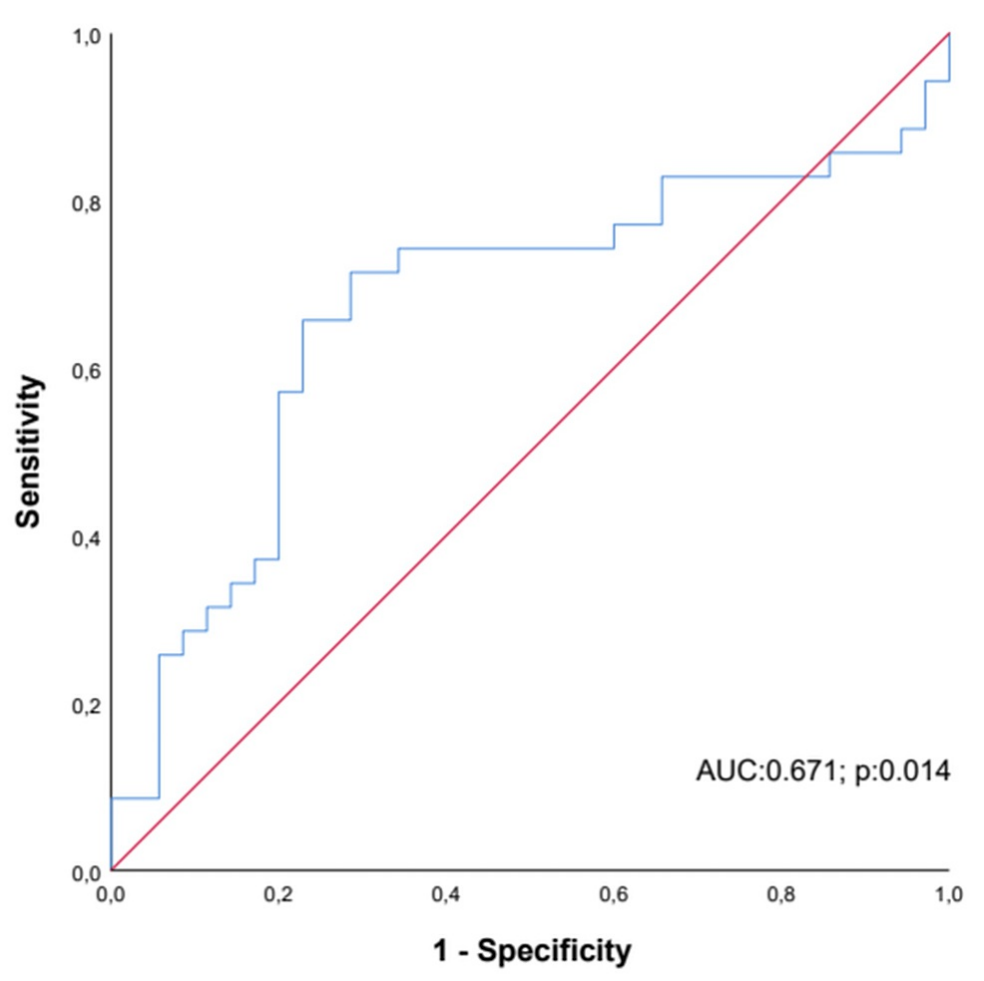
ROC curve for diagnostic performance of ADC to discriminate diabetic patients. ROC: receiver-operating characteristic; ADC: apparent diffusion coefficient

The median pancreatic ADC values before treatment in diabetic patients were 1.39 (min-max: 1- 2.40) × 10-3 mm2/s. In the control group, it was 1.62 (min-max: 1.22-2.11) × 10^−3^ mm^2^/s. A significant difference was found between the control and patient groups in the median pancreatic ADC values (p = 0.014). When the optimum cut-off value of ADC was taken at 1.53 × 10−3 mm2/s to differentiate diabetic patients from the control group, sensitivity was 71.4%, and specificity was 71.5% (AUC: 0.671; 95 CI%: 0.538-0.804; p<0.014) (Figure 1).

There was a significant difference between the pancreatic head, body, and tail ADC values of the diabetic patient and control groups before treatment (p = 0.011, p = 0.026, and p = 0.010, respectively) (Table 3).

**Table 3:** ADC values of the control and diabetic groups before treatment ADC: apparent diffusion coefficient; min: minimum; max: maximum

	Control Group			Diabetic Group			p values
	Median	Min	Max	Median	Min	Max	
ADC head (mm^2^/s × 10^−3^)	1.61	1.25	2.12	1.49	1.11	2.57	0.011
ADC body (mm^2^/s × 10^−3^)	1.69	1.27	2.23	1.49	0.99	2.43	0.026
ADC tail (mm^2^/s × 10^−3^)	1.53	1.07	1.99	1.26	0.90	2.20	0.010

The median pancreatic ADC values of diabetic patients after treatment were 1.57 (min-max: 1.33-2.57) × 10-3 mm2/s. There was a significant difference between the median pancreatic ADC values of diabetic patients before and after treatment (p<0.001).

The differences between pancreatic head, body, and tail ADC values before and after treatment were statistically significant(p<0.001, for all).

There was no significant difference between the median pancreatic ADC values of the control group and the diabetic patients post-treatment (p = 0.842).

**Figure 2:**
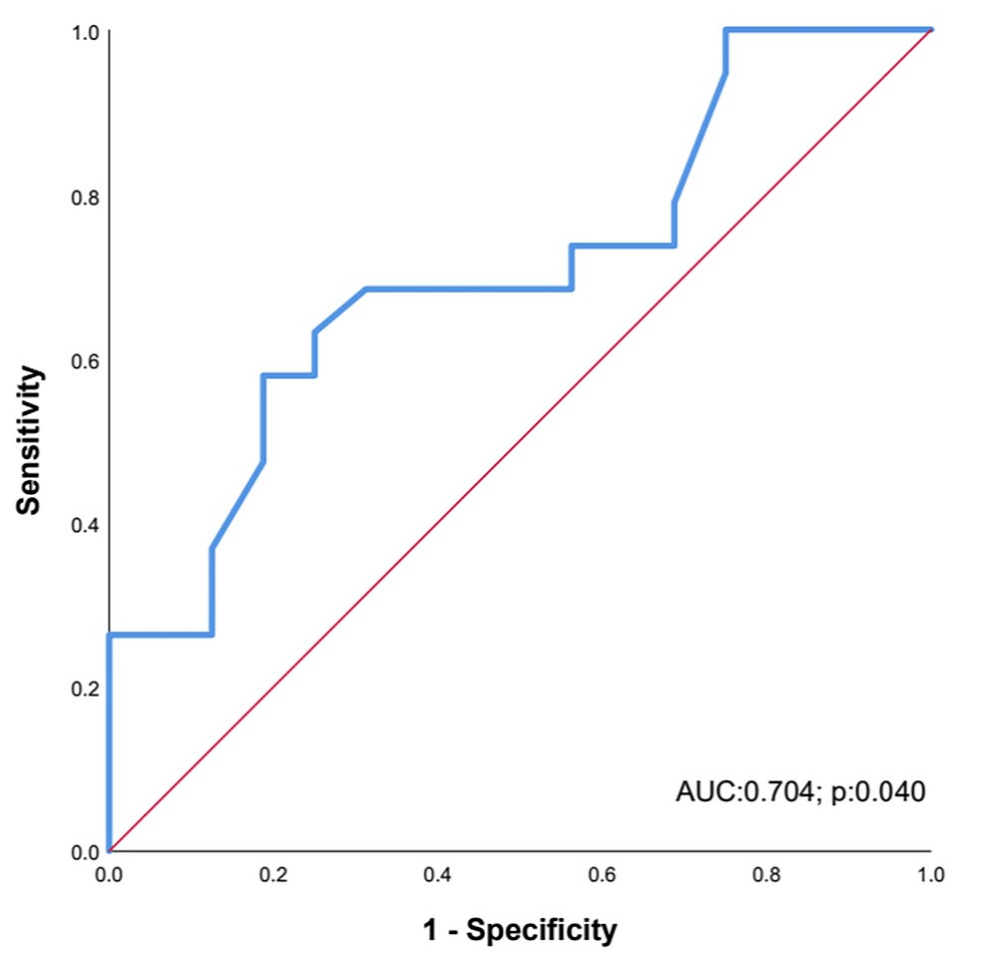
ROC curve for diagnostic performance of ADC tail difference to evaluate the response of treatment. ROC: receiver-operating characteristic

According to ADA guidelines, the cut-off value HbA1c <6.5 was accepted to evaluate response to treatment. The change in pancreatic tail level ADC over 0.215 x 10^−3^ mm^2^/s had 63% sensitivity and 75% specificity in evaluating response to treatment (AUC: 0.704; p=0.040; 95 CI%: 0.530-0.878) (Figure 2).

There was a negative correlation between the ADC and HbA1c values of the patients (rho:- 0.298; p=0.012). It was determined that pancreatic ADC values increased significantly with age (rho: 0.761; p<0.001). In addition, there was a low level of significant negative correlation between e-GFR and ADC values (rho: 0.339; p=0.046).

## Discussion

In diabetic patients, due to hyperglycemia, deposits occur in the pancreatic islet associated with progressive cell loss and pancreatic fibrosis, which is claimed to have a considerable effect on the pathogenesis of glucose intolerance [14].

In previous studies, it has been reported that with the measurement of ADC on diffusion-weighted MR images, abnormal histopathological changes, including fibroblast expression, can be detected and monitored during the progression of fibrosis in the pancreas and kidney [15]. Insulin and metformin can be used in combination to treat early diagnosed type II diabetes patients. The response of patients to treatment is evaluated by laboratory parameters such as fasting blood glucose and HbA1c [13].

Although imaging parameters that can be used to evaluate histopathological changes in the pancreas due to glucose toxicity in diabetic patients have been defined in the literature, there is no study evaluating their use in the evaluation of response to treatment. To the best of our knowledge, this is the first study in which response to treatment was evaluated with diffusion-weighted MRI in patients with newly diagnosed type II diabetes. Studies have reported that the prevalence of type 2 diabetes is higher in males [1,16]. Similarly, the majority of newly diagnosed diabetes patients in the study were male. Noda et al. reported a negative correlation between HbA1c and ADC [12]. The study showed a similar correlation in diabetic patients, although it also included post-treatment HbA1c. Watanabe et al. reported that the mean ADC value decreased continuously as the degree of pancreatic fibrosis progressed [11]. Noda et al. reported a lower ADC value in diabetic patients in their study comparing diabetic and non-diabetic patients [12]. The same, we found that diabetic patients had lower pre-treatment ADC values compared to the control group.

Metformin has been recognized in recent years as the most appropriate initial therapy for patients with type 2 diabetes in various guidelines, including that of the European Association for Diabetes Research and the ADA [17]. It has been reported that insulin therapy is usually started a few years after the onset of the disease in patients with T2DM, an earlier onset would be better, and that early intensive insulin therapy had a more favorable result than conventional therapy in terms of preserving β-cell function in newly diagnosed subjects [18]. The study found that ADC values increased after treatment with insulin + metformin compared to before treatment. We think that this is due to decreased fibrosis and preservation of β-cells. Further clinical trials will be required to confirm our results and evaluate response to treatment.

It has been stated in the literature that increasing extracellular matrix secondary to pancreatic atrophy with age causes an increase in diffusion. Noda et al. found the mean ADC value to be 2.58±0.85 × 10^−3^ mm^2^/s in non-diabetic individuals with a mean age of 63 years, and 1.30 × 10^−3^ mm^2^/s in non-diabetic individuals with a mean age of 52 years, and Şahan et al. found a positive correlation between age and ADC [12,19]. In the study, the mean ADC value was 1.62 × 10^−3^ mm^2^/s in non-diabetic patients with a mean age of 53, and there was a similar correlation.

Pancreatic ADC measurements were generally calculated by the average measurements made from the head, body, and tail levels in the literature [19,20]. The diabetic patients and the control group measurements were made from these parts of the study, but each part was evaluated separately, unlike the literature. We determined that the measurements made from the tail were more significant than the others in evaluating the response to treatment. We think this may be related to the fact that the tail segment is more cellular and more responsive to treatment.

In studies on renal and pancreatic fibrosis detectability in the literature, different b-values (50, 400, 800, 1000, 1500 s/mm^2^) were used instead of standard b-values in DWI [15,20]. The b value we preferred in the study was 50 and 800 s/mm^2^. Prospective studies are needed on the relationship between the b value and the detectability of pancreatic fibrosis.

Among the limitations of our study were the following: first, this was a single-center study with a relatively small number of patients. Second, assessments were made by only one observer. Third, only two b-values (50 and 800 mm^2^/s) were used to calculate the ADC. Fourth, the reproducibility of the ADC values was not evaluated.

## Conclusions

Pancreatic ADC values can be used to distinguish diabetic patients from healthy subjects. In pancreatic ADC values; there is a positive correlation with increasing age and a negative correlation with HbA1c and eGFR. Pancreatic ADC values of newly diagnosed type II diabetes patients treated with insulin + metformin can be used as a marker of pancreatic function in the evaluation of response to treatment and clinical decisions.
